# Volumetric Modulated Arc Therapy for High-Risk and Very High-Risk Locoregional Prostate Cancer in the Modern Era: Real-World Experience from an Asian Cohort

**DOI:** 10.3390/cancers16172964

**Published:** 2024-08-25

**Authors:** Qijun Du, Kuen Chan, Michael Tsz-Yeung Kam, Kelvin Yu-Chen Zheng, Rico Hing-Ming Hung, Philip Yuguang Wu

**Affiliations:** Department of Clinical Oncology, Pamela Youde Nethersole Eastern Hospital, Hong Kong, China; chank1@ha.org.hk (K.C.); kty231@ha.org.hk (M.T.-Y.K.); zky835@ha.org.hk (K.Y.-C.Z.); hhm050@ha.org.hk (R.H.-M.H.)

**Keywords:** high-risk locoregional prostate cancer, volumetric modulated arc therapy, whole pelvic radiotherapy, simultaneous integrated boost, PSMA PET-CT

## Abstract

**Simple Summary:**

Definitive radiotherapy (RT) with androgen deprivation therapy is the standard of care for high- or very high-risk locoregional prostate cancer. Despite various randomized trials, the optimal RT strategy is undefined. Practices in the selection for whole pelvic radiotherapy (WPRT), RT doses and techniques are considerably heterogeneous. Image-guided radiotherapy (IGRT) techniques have emerged to allow for precise dose prescription. The current study reports on the 5-year institutional experience of definitive volumetric modulated arc therapy using a standardized strategy of WPRT based on the estimated nodal risk, as well as simultaneous integrated boost to the involved nodes in the context of contemporary staging and RT techniques. An analysis of 209 patients demonstrated excellent clinical outcomes. Initial PSMA PET-CT compared to conventional imaging staging was associated with better biochemical relapse-free survival in patients with ISUP grade group 5 disease. These results suggest the approach of modern IGRT and the risk-adapted use of PSMA PET-CT staging and nodal irradiation in real-world clinical practice.

**Abstract:**

This study retrospectively evaluates the clinical outcomes of definitive volumetric modulated arc therapy (VMAT) for high-risk or very high-risk locoregional prostate cancer patients from an Asian institution. Consecutive patients who received VMAT (76 Gy in 38 fractions) between January 2017 and June 2022 were included. Whole pelvic radiotherapy (WPRT) (46 Gy in 23 fractions) was employed for clinically node-negative disease (cN0) and a Roach estimated risk of ≥15%, as well as simultaneous integrated boost (SIB) of 55–57.5 Gy to node-positive (cN1) disease. The primary endpoint was biochemical relapse-free survival (BRFS). Secondary endpoints included radiographic relapse-free survival (RRFS), metastasis-free survival (MFS) and prostate cancer-specific survival (PCSS). A total of 209 patients were identified. After a median follow-up of 47.5 months, the 4-year actuarial BRFS, RRFS, MFS and PCSS were 85.2%, 96.8%, 96.8% and 100%, respectively. The incidence of late grade ≥ 2 genitourinary (GU) and gastrointestinal (GI) toxicity were 15.8% and 11.0%, respectively. No significant difference in cancer outcomes or toxicity was observed between WPRT and prostate-only radiotherapy for cN0 patients. SIB to the involved nodes did not result in increased toxicity. International Society of Urological Pathology (ISUP) group 5 and cN1 stage were associated with worse RRFS (*p* < 0.05). PSMA PET-CT compared to conventional imaging staging was associated with better BRFS in patients with ISUP grade group 5 (*p* = 0.039). Five-year local experience demonstrates excellent clinical outcomes. PSMA PET-CT staging for high-grade disease and tailored pelvic irradiation based on nodal risk should be considered to maximize clinical benefit.

## 1. Introduction

Globally, prostate cancer is the second most frequent cancer among men [1]. In Hong Kong, it is the third most frequent cancer and the fourth leading cause of cancer deaths in men [2]. Although the majority of patients present without distant metastasis, high-risk and very high-risk locoregional disease represent a significant proportion of prostate cancer burden locally [3,4]. More than one-third have high grade histology (Gleason score ≥ 8) and, in 39.2% of cases, prostate specific antigen (PSA) > 20 µg/L at the time of diagnosis [3].

Patients with high-risk or very high-risk locoregional prostate cancer are recommended to undergo definitive radiotherapy (RT) in combination with androgen deprivation therapy (ADT) for 1.5 to 3 years [5,6,7]. Contemporary RT doses of 74–81 Gy to the prostate (equivalent dose at 2 Gy per fraction, α/β ratio = 1.5 Gy) produce 5-year biochemical relapse-free survival (BRFS) and cancer-specific survival in the order of 60–95% and 87–98%, respectively [8,9,10,11,12,13,14]. However, the role of elective irradiation of the pelvic lymphatics has been a subject of ongoing debate. The risk of pelvic nodal involvement is estimated clinically using the Roach formula [15], and elective nodal irradiation is typically advocated for those with a significant risk (≥15–20%) [16,17,18,19]. Early randomized trials using conventional RT doses and techniques failed to demonstrate clear clinical benefits of whole pelvic RT (WPRT) over prostate-only radiotherapy (PORT) [16,17,18]. More recently, the phase III POP-RT trial showed significant advantage in 5-year biochemical failure-free survival (BFFS), disease-free survival (DFS) and distant metastasis-free survival (MFS) but not overall survival (OS), being in favor of WPRT in high-risk or very high-risk clinical node-negative (cN0) prostate cancer [19]. However, it is noteworthy that the POP-RT trial represented a uniformly higher-risk cohort, with over half of the patients belonging to the National Comprehensive Cancer Network (NCCN) very high-risk group, and median Roach nodal risk of 37.8% [19]. 

On the other hand, there is greater consensus regarding the delivery of primary WPRT for patients with clinically node-positive disease (cN1) [20,21]. Large-scale population-based observational studies and retrospective cohorts suggest potential survival benefits from initial radiotherapy in patients with node-positive disease [22,23,24,25]. In addition, an analysis of cN1 patients from the STAMPEDE trial also provides evidence to support the role of WPRT in cN1 prostate cancer [26].

In the past, barriers to the extensive adoption of WPRT were attributed to concerns of a higher incidence of radiation-related toxicities compared to PORT, in particular gastrointestinal (GI), genitourinary (GU) and hematological events [18,27,28,29,30]. However, data from earlier studies were conflicting, and most utilized conventional radiation techniques. In the recent decade, radiotherapy for prostate cancer has evolved to intensity-modulated radiation therapy (IMRT) or volumetric modulated arc therapy (VMAT) being adopted as the standard of care in most parts of the world, with highly precise RT delivery under image guidance to allow for dose escalation while minimizing treatment-related toxicity [31,32,33,34]. Furthermore, VMAT simultaneous integrated boost (SIB) is feasible for highly conformal doses to individual involved nodes [35,36]. This approach allows for the timely delivery of similar tumoricidal doses to clinically positive nodes as the prostate primary, and has drawn increasing interest in attempts to maximize locoregional control and survival outcomes. 

Accurate staging and risk stratification in locoregional prostate cancer can be challenging. Novel imaging modalities such as prostate-specific membrane antigen positron emission tomography–computed tomography (PSMA PET-CT) have been increasingly available, offering greater sensitivity and specificity in the detection of regional and distant metastases compared to conventional imaging [37,38,39]. The detection of regional nodes or oligo-metastasis will likely increase in the patient population with high-risk or very high-risk disease and impact the staging and treatment decisions. Nevertheless, the role of PSMA PET-CT in initial staging and the consequent change in the treatment strategy on clinical outcomes has not been characterized. 

Due to the lack of conclusive evidence on the optimal strategy, the management of high-risk and very high-risk locoregional prostate cancer is considerably heterogeneous across the world. Practices in disease staging, selection for WPRT, volume definition, prescription doses, and the use of androgen deprivation therapy (ADT) vary substantially. From the best of our knowledge, reports on treatment outcomes of definitive RT for locally advanced prostate cancer in the Chinese population are scarce. The current study aims to report our 5-year local experience for high-risk and very high-risk locally advanced prostate cancer in the era of contemporary staging and radiotherapy techniques, including oncological outcomes and treatment-related toxicity.

## 2. Materials and Methods

### 2.1. Patients

Consecutive patients with histologically confirmed, pelvis-confined high-risk or very high-risk locoregional prostate cancer who received definitive radiotherapy with neoadjuvant–concurrent–adjuvant ADT with or without WPRT at the Department of Clinical Oncology of Pamela Youde Nethersole Eastern Hospital between 1 January 2017 and 30 June 2022 were retrospectively reviewed in this study.

The patients were staged according to the American Joint Committee on Cancer (AJCC) TNM staging 8th Edition [40]. Pelvis-confined prostate cancer refers to high-risk or very high-risk cN0 prostate cancer according to the NCCN guideline [20], or cN1 disease. Clinically positive pelvic node was defined by enlarged lymph node(s) (≥1 cm in short axis) within the true pelvis on staging magnetic resonance imaging (MRI) or CT, the presence of suspicious morphology or positive uptake on PSMA PET-CT [41]. Patients with positive lymph nodes beyond the true pelvis, distant metastasis, prior history of prostatectomy, RT prescribed with palliative intent or unable to complete the intended RT treatment were excluded.

### 2.2. Radiotherapy

Patients were simulated in the supine position on a customized vacuum bag or alpha cradle and instructed to maintain a comfortably full bladder and empty rectum. Simulation CT images with 3 mm slice thickness were obtained. Intravenous contrast was administered for patients intended for WPRT. Co-registration of simulation CT with diagnostic multiparametric MRI and PSMA PET-CT (if available) was performed for target delineation. 

The Primary Tumor Clinical Target Volume (CTVp) encompassed the whole prostate and any extra capsular tumor extension, as well as the bilateral seminal vesicles. For patients with a Roach estimated nodal risk of ≥15%, the Elective Nodal Clinical Target Volume (CTVe) included the bilateral common iliac region (from the aortic bifurcation), internal and external iliac, presacral and obturator regions as per recommendations by the Radiation Therapy Oncology Group (RTOG) [42]. For patients with clinically involved nodes at the time of RT that required SIB, they were individually contoured as Nodal Gross Tumor Volume (GTVnx), and 3 mm circumferential margin was applied to form individual Nodal Clinical Target Volumes (CTVnx). The Primary Tumor Planning Target Volume (PTVp) was generated by applying a 1 cm margin to CTVp, being 0.5 cm in the posterior direction. The Nodal Planning Target Volumes (PTVe and PTVnx) were generated by applying a 0.5 cm circumferential margin to the respective CTV. 

The radiotherapy dose was 76 Gy in 38 daily fractions to the PTVp. In patients receiving WPRT, 46 Gy was prescribed to the PTVp and PTVe in the first 23 fractions, followed by an additional 30 Gy in 15 daily fractions to PTVp in a sequential manner. SIB to individual involved nodes were prescribed 55–57.5 Gy in the initial 23 fractions to achieve an equivalent dose at 2 Gy per fraction (EQD2) of 60–66 Gy (α/β ratio = 1.5), in addition to the usual elective dose to the lymphatic regions. 

The VMAT was delivered on TruebeamTM (Varian Medical Systems, Palo Alto, CA, USA) using 6 MV photon. Image guidance consisted of online cone-beam CT verification complemented by 6-degrees-of-freedom couch corrections. Planning objectives and dose specifications for tumor targets and organs at risk (OARs) are described in Appendix A. 

Our institutional policy recommends neoadjuvant, concurrent and adjuvant ADT in the form of luteinizing hormone-releasing hormone analogue (LHRHa) for a total duration of 3 years. Radiotherapy was delivered 10 to 12 weeks after the first dose of ADT. 

### 2.3. Follow-Up

Patients underwent regular follow-up according to institutional protocol, which occurred at 2 to 4 weeks and 3 months after completion of RT, then every 3–6 months for the first 5 years and annually thereafter. Clinical assessments of disease status and treatment-related effects were performed at each visit. Routine PSA check was performed starting at 3–6 months post-RT. More frequent follow-ups occurred for individual patients per clinician discretion. 

Biochemical relapse was defined using the American Society for Radiation Oncology (ASTRO) definition (nadir PSA plus three consecutive PSA rises with backdating the time to failure, being the midpoint between the dates of the PSA nadir and the first PSA rise) [43]. Radiographic relapse was defined by the first evidence of locoregional or distant relapse using radiological assessment (CT, MRI, bone scan or PSMA PET-CT) upon biochemical failure or clinical suspicion. Metastatic relapse was defined by the first radiologically confirmed distant metastasis. 

Radiotherapy-related toxicities were evaluated using the Common Terminology Criteria for Adverse Events version 5.0 [44]. Acute toxicities were defined as those occurring during or within 3 months of RT completion and late toxicities as those manifesting thereafter. 

### 2.4. Endpoints and Statistical Analysis

The primary endpoint was BRFS per ASTRO definition, while secondary endpoints included radiographic relapse-free survival (RRFS), MFS, prostate cancer-specific survival (PCSS), failure patterns and radiotherapy-related toxicity. Survival outcome measures were determined from the date of RT initiation to the occurrence of the first failure event. Patients were censored at their last follow-up or death. Kaplan–Meier estimates on the probability of clinical outcomes at 24, 36, 48 and 60 months were derived. Failure patterns at the time of the first radiographic failure were analyzed. To compare treatment toxicities and relevant variables across subgroups, the Chi-square test or Fisher’s exact test were used. The associations between clinical outcomes and the relevant clinicopathological factors, staging method and treatment variables were tested using a Cox proportional hazards regression model, with significant variables from univariate analysis entered into multivariate analysis. A *p*-value of <0.05 indicated statistical significance. All statistical analyses were conducted using SPSS (IBM, Statistics Version 26).

## 3. Results

### 3.1. Clinicopathological and Treatment Characteristics

A total of 209 consecutive patients underwent definitive VMAT during the study period. The median age at diagnosis was 72 years (interquartile range (IQR) 67–77), with the vast majority with an Eastern Cooperative Oncology Group (ECOG) performance status of 0 or 1 (88%).

Systematic prostate biopsy was conducted in 163 patients (78%), while additional image-guided targeted biopsy was performed in 28 patients (13.4%). Transrectal was the predominant route (79.4%), while transperineal (TP) prostate biopsy was performed in 27 patients (12.9%). Initial staging involved PSMA PET-CT for 89 patients (42.6%).

The median baseline PSA was 22.6 ng/mL (IQR 11.4–42.7). Ninety-one patients (43.6%) had International Society of Urological Pathology (ISUP) grade group 4 or 5 disease. The median Roach estimated nodal risk was 31% (IQR 22.3–43.9%). In terms of T stage, 71 patients (34%) had cT3a disease, while 51 patients (24.4%) had T3b or above. Ninety-seven (46.4%) and ninety-six patients (45.9%) had NCCN high-risk and very high-risk cN0 disease, respectively. Sixteen patients (7.7%) had cN1 disease. 

A total of 99% of patients received or were planned to receive at least 2 years ADT. Ten patients (4.8%), mostly those with cN1 disease, received ADT for longer than 3 years. Two patients (1%) received ADT for less than 2 years due to medical comorbidities or refusal. 

All patients completed definitive VMAT as planned. One hundred and sixty-one (77%) patients received WPRT. Among patients with cN1 disease (N = 16), all of them received WPRT, and 12 of them received additional SIB to the involved pelvic lymph nodes. The median overall treatment time (OTT) from initiation to completion of RT was 55 days (IQR 52–56). 

For details on clinicopathological and treatment characteristics, refer to Table 1. 

### 3.2. Treatment Efficacy, Failure Patterns and Toxicity

The median follow-up duration was 47.5 months (IQR 32.5–65.1). Actuarial estimates for BRFS, RRFS, MFS and PCSS at 2-years and 4-years were 93.8%, 99.5%, 99.5% and 100% and 85.2%, 96.8%, 96.8% and 100%, respectively (Table 2). For patients with cN1 disease (N = 16), the 2-year BRFS, RRFS, MFS and PCSS were 78.8%, 93.8%, 93.8% and 100%, respectively.

A total of five patients developed radiographic relapse during the follow-up period. Three of these patients had cN1 disease at initial diagnosis. All these patients underwent definitive WPRT, and two of them received SIB to the involved nodes. The predominant failure pattern at first recurrence was distant (bone metastasis), with concomitant regional nodal failure in one patient. One patient experienced isolated local failure. The median time to any radiographic failure was 38.8 months (range 19.5–55.6). All relapsed patients received subsequent palliative systemic treatment, including the re-initiation of ADT, and docetaxel or androgen receptor signaling inhibitors. Table 3 elaborates on the clinicopathological and treatment characteristics for each patient who developed radiographic relapse.

Overall, 64 (30.6%) patients developed grade ≥ 2 RT-related acute GU toxicity, with 15 (7.2%) of them being grade 3. Eighteen (8.6%) patients developed grade 2 acute GI toxicity. No grade 3 acute GI toxicity was observed. Regarding late toxicity, the incidence of grade ≥ 2 GU and GI toxicity occurred in 33 (15.8%) and 23 (11.0%) patients, respectively. The incidences of grade 3 GU and GI toxicity were low (four patients, 1.9% for each event). No grade 4 or above RT-related toxicity occurred. One patient (0.5%) developed symptomatic sacral insufficiency fracture and was managed conservatively. For detailed information, see Table 4.

### 3.3. Role of WPRT on Clinical Outcomes and Toxicity

**Figure 1:**
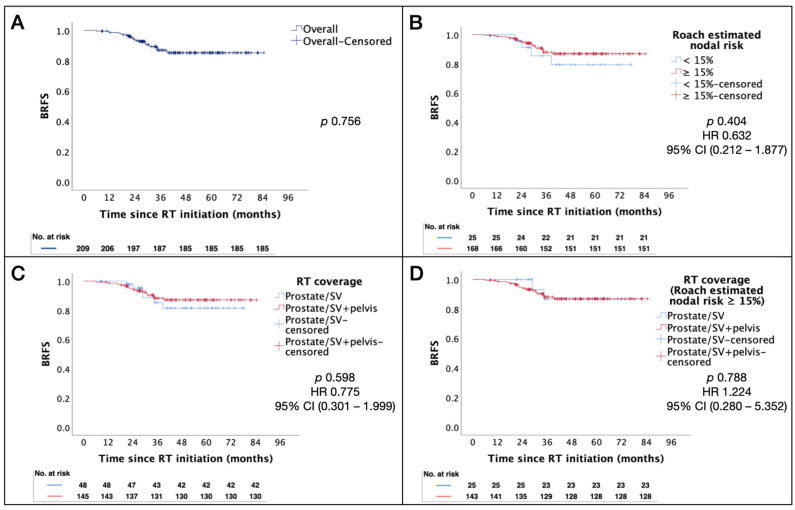
Kaplan–Meier estimates of BRFS by RT coverage and Roach nodal risk. (**A**) Overall. (**B**) cN0M0 patients stratified by Roach estimated nodal risk. (**C**) cN0M0 patients stratified by RT coverage. (**D**) cN0M0 patients with Roach estimated nodal risk ≥ 15% stratified by RT coverage. Abbreviations: BRFS = biochemical relapse-free survival; RT = radiotherapy; ASTRO = American Society for Radiation Oncology; SV = seminal vesicle; HR = hazard ratio; CI = confidence interval.

The Kaplan–Meier estimates for BRFS in patients with high- or very-high risk cN0 prostate cancer were similar in patients who had a Roach formula estimated nodal risk of ≥15% compared to those with lower nodal risk (*p* = 0.404). In addition, no significant difference in BRFS was found in patients who received WPRT versus PORT (*p* = 0.598) in the overall population, and in the subgroup of patients with a Roach estimated nodal risk of ≥15% (see Figure 1A–D for Kaplan–Meier estimates of BRFS, stratified by RT coverage and Roach estimated nodal risk). Upon examination of the underlying clinicopathological characteristics between RT disposition, it was noted that patients who received WPRT also tended to exhibit a significantly higher number of other adverse prognostic features, including a higher positive core-to-total core ratio (*p* = 0.015) and higher ISUP grade group 4–5 core-to-total core ratio (*p* = 0.019) (Table 5). 

In terms of RT-related toxicity, a higher incidence of grade 2 acute GI toxicity was observed in patients who received WPRT compared to PORT, although this was not statistically significant (11.4% vs. 2.1%, *p* = 0.079). Otherwise, no significant difference in grade ≥ 2 acute or late toxicity was observed between the patient groups (refer to Table 4 for details on the incidence of RT-related toxicity by RT disposition). 

In the small subgroup of patients with cN1 disease (N = 16), the prescription of additional SIB did not result in a significant difference in BRFS (*p* = 0.956) compared to elective-dose WPRT alone. On the other hand, the toxicity profile was also similar.

### 3.4. Assessment of Prognostic Factors on Clinical Outcomes 

Table 6 summarizes the associations of clinicopathological variables on BRFS and RRFS. ISUP grade group 5 disease and cN1 stage were associated with significantly worse RRFS (*p* < 0.05 on both univariate and multivariate analysis). Notably, the impact of PSMA PET-CT staging on BRFS was marginally significant in the univariate analysis (hazard ratio (HR) 0.428, *p* = 0.083, 95% confidence interval (CI) 0.159–1.150). 

### 3.5. Impact of PSMA PET-CT Staging 

The use of PSMA PET-CT staging at baseline was significantly associated with the presence of adverse clinicopathological features such cN1 stage (15 patients staged using PSMA PET-CT vs. 1 patient using conventional imaging), the presence of ISUP grade group 5 disease (24 patients staged using PSMA PET-CT vs. 9 patients using conventional imaging), and a higher Roach estimated nodal risk (median 37.2% in patients staged using PSMA PET-CT vs. 26.3% in patients staged using conventional imaging). 

**Figure 2:**
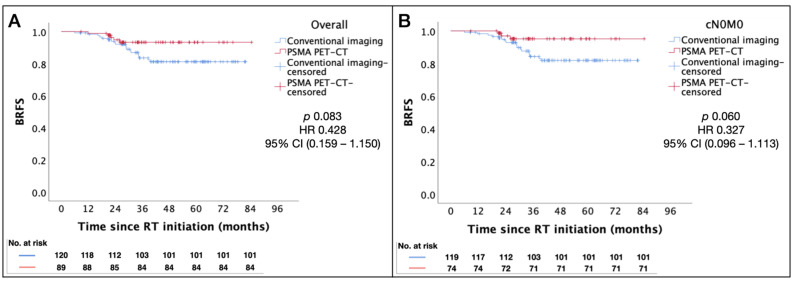
Kaplan–Meier estimates of BRFS by staging modality. (**A**) Overall. (**B**) cN0M0 patients. Abbreviations: BRFS = biochemical relapse-free survival; ASTRO = American Society for Radiation Oncology; PSMA PET-CT = prostate-specific membrane antigen positron emission tomography–computed tomography; RT = radiotherapy; HR = hazard ratio; CI = confidence interval.

Staging with PSMA PET-CT was associated with a trend towards better BRFS in the overall patient cohort, being more apparent in those with cN0 disease, although this did not reach statistical significance (*p* = 0.083 and 0.060, respectively). Figure 2A,B illustrate the Kaplan–Meier estimates for BRFS using conventional imaging vs. PSMA PET-CT staging. 

**Figure 3:**
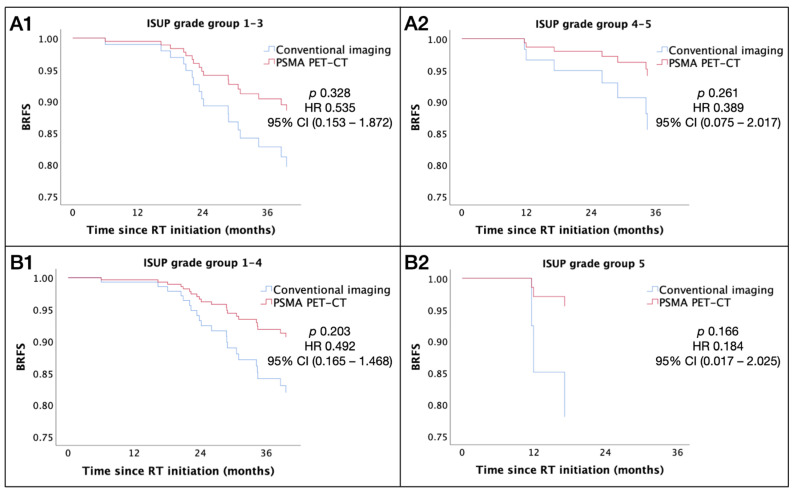
BRFS by staging modality and ISUP grade group. (**A1**) ISUP grade group 1–3. (**A2**) ISUP grade group 4–5. (**B1**) ISUP grade group 1–4. (**B2**) ISUP grade group 5. Abbreviations: BRFS = biochemical relapse-free survival; ISUP = International Society of Urological Pathology; PSMA PET-CT = prostate-specific membrane antigen positron emission tomography–computed tomography; RT = radiotherapy; HR = hazard ratio; CI = confidence interval.

To further identify a subgroup of patients where initial PSMA PET-CT staging may impact the clinical outcome, the association of the staging modality with BRFS was evaluated, accounting for interactions with other relevant clinicopathological variables using the Cox proportional hazards model. Patients who had initial PSMA PET-CT staging trended towards better BRFS, and this association was more prominent with higher ISUP grade disease (*p* = 0.039) (Figure 3 and Table 7). In patients with ISUP grade group 5 disease, there was a strong correlation for clinical benefit in BRFS, in favor of PSMA PET-CT over conventional imaging (Table 7). 

## 4. Discussion

In this study, we reported the clinical outcomes of 209 consecutive patients with high-risk or very high-risk locoregional prostate cancer treated with definitive VMAT with standard long-term ADT administered for 3 years. After a median follow-up of 47.5 months, we observed excellent BRFS of 85.2% by the ASTRO definition, RRFS and MFS of 96.8%, PCSS of 100% at 4 years and a low incidence of grade ≥ 2 late toxicities. The results suggest favorable clinical outcomes given the relatively poor risk profile of our patient cohort: 45.9% belonged to the NCCN very high-risk group, 7.7% had cN1 disease, and the median Roach estimated nodal risk was 31%. To the best of our knowledge, this is one of the largest reports on definitive RT for Chinese patients with locoregionally advanced prostate cancer in the context of contemporary image-guided radiotherapy (IGRT) techniques, and it contributes to real-world clinical evidence. 

The standard treatment for high-risk or very high-risk cN0 prostate cancer is definitive RT with long-term ADT for 1.5 to 3 years [45,46,47]. The role of WPRT to PORT, however, has been an unresolved question for more than two decades. Two randomized trials from the nineties explored the value of adding WPRT for localized prostate cancer. The RTOG 9413 trial recruited 1323 intermediate- or high-risk prostate cancer patients with a Roach estimated nodal risk of ≥15% and used a 2 × 2 factorial design to compare WPRT versus PORT as well as the timing of ADT (neoadjuvant or adjuvant) [16,17,18]. The long-term results demonstrated no significant survival benefit of WPRT over PORT [16,17,18]. The GETUG-01 trial randomly assigned 446 patients with T1b-T3N0M0 prostate cancer to WPRT or PORT, and the patients received 4–8 months of ADT. No difference in event-free survival (EFS) or OS was observed after more than 11 years of follow-up [27,48]. More recently, the phase III POP-RT study using contemporary moderately hypofractionated IMRT demonstrated clinical benefits of WPRT over PORT for patients with high-risk or very high-risk cN0 prostate cancer with an estimated nodal risk of ≥20% [19]. A significant improvement in BFFS, DFS and distant MFS, but not OS, was demonstrated, in favor of WPRT [19]. Furthermore, an analysis of 28724 men with unfavorable intermediate-risk or high-risk prostate cancer from the National Cancer Database demonstrated that WPRT correlated with improved OS in men with Gleason 9 and 10 disease or a Roach estimated nodal risk of ≥10% [49]. While cross-study comparisons are fraught with biases, it is noteworthy that the randomized trials discussed above vary considerably in patient inclusion, the method of tumor staging, RT volumes, doses and techniques and the duration of ADT. Potential reasons for the positive clinical benefit of WPRT demonstrated in the POP-RT trial include a relatively high-risk cohort (46.4% of patients had T3b-T4 tumors, 49.1% had Gleason 8–10 disease and the median Roach estimated nodal risk was 37.8%), higher prescription doses to the elective pelvis (50 Gy) and the inclusion of the common iliac region within the nodal CTV [19]. In addition, around 80% of patients had initial tumor staging using PSMA PET-CT to exclude patients with nodal or distant metastasis, which is not easily detected using conventional imaging [19]. Although our study included patients with a similar poor-risk profile, no significant difference in the treatment outcome was observed in patients who received WPRT versus PORT. This could be due to the overall smaller patient numbers with a limited number of events and the significant imbalance in patient numbers between the treatment groups. On the other hand, it is worthwhile to note that in our study, patients who received WPRT had in general poorer-risk disease (such as a higher proportion of high-grade biopsy cores). In addition, no significant difference in grade ≥ 2 toxicity was observed in patients who received WPRT compared to PORT. Table 8 summarizes the characteristics and outcomes of the WPRT subgroup of randomized trials and that of our current study. The RTOG 0924 and PIVOTALboost trials will further shed light on the role of contemporary WPRT in patients with unfavorable intermediate- and favorable high-risk disease profiles [50,51]. 

For patients with cN1 disease, adding definitive WPRT to ADT significantly improves survival, demonstrated by large-scale retrospective and population-based observational studies, and is considered the standard of care [20,22,23,24,25,26]. A more recent retrospective study of 51 consecutive patients with cN1 disease showed that dose escalation to the involved pelvic lymph nodes to higher than 60 Gy in EQD2 improved BRFS, progression-free survival (PFS) and MFS at 4 and 7 years [52]. Extrapolating from the clinical benefit derived from dose escalation to the prostate primary [53,54] and the experience from other cancer types [55], dose escalation to the involved pelvic lymph nodes using modern radiotherapy techniques may offer advantages in disease control. In the small subgroup of patients with cN1 disease in the current study, VMAT SIB to the involved pelvic lymph nodes to doses of 60–66 Gy (EQD2) with stringent planning objectives and image guidance resulted in reasonable treatment outcomes without increases in toxicity. In addition, the 2-year RRFS and MFS (93.8%) of our current cN1M0 cohort was slightly better than the cN1 cohort of the STAMPEDE trial (2-year FFS 89%), for which boost to the involved pelvic nodes was not adopted [26]. Nonetheless, the role of dose escalation to the involved nodes requires further validation by prospective trials. 

Patients with high-risk or very high-risk locoregional prostate cancer are prone to systemic relapse. The rate of metastatic recurrence in this subgroup of patients was reported to be approximately 10–40% at 5 years after primary treatment [4]. In our study, the predominant radiographic failure pattern was distant metastasis, occurring in four out of five patients with radiographic relapse, with concurrent regional failure in one patient. Notably, three of the five patients had cN1 disease at diagnosis. This calls for the need for treatment intensification to enhance systemic control. Randomized trials have indicated that adding long-term ADT to RT significantly prolonged MFS and OS, with 10-year absolute benefits of 8.6% and 7.7%, respectively [56]. The addition of adjuvant anti-androgen abiraterone to long-term ADT and definitive RT in cases of high-risk cN0 or cN1 disease has been supported by the STAMPEDE trial, demonstrating significantly improved MFS and OS, albeit with increased treatment-related toxicity [57]. Furthermore, the GETUG-AFU-23 trial is evaluating the role of neoadjuvant cabazitaxel and pelvic radiotherapy in patients with unfavorable high-risk prostate cancer, and the results are eagerly awaited [58]. 

Several studies have established a dose-response relationship with cancer outcomes in high-risk localized prostate cancer [8,9,10,11,12,13,14]. Conventional dose fractionation was employed in the current study. In recent years, hypofractionation has become a subject of increasing interest, harnessing the radiobiological advantages in prostate cancer radiotherapy. The introduction of precision RT techniques, such as VMAT and adaptive IGRT, enable isotoxic dose escalation and improvements in the therapeutic ratio. The results of the phase III FLAME trial supported the advantage of an intraprostatic focal boost of up to 95 Gy using the IMRT SIB technique in biochemical DFS, without a significant increase in toxicity or adverse impact on the quality of life [54]. The hypoFLAME study further demonstrated that stereotactic body radiation therapy (SBRT) with ultra-hypofractionated doses of 35 Gy in 5 weekly fractions to the whole prostate gland, with isotoxic boost up to 50 Gy to multiparametric MR-defined tumors, was associated with acceptable acute GU and GI toxicity [53]. Long-term results from these studies to elucidate the effects of isotoxic dose escalation to the primary prostate tumor on cancer outcomes is eagerly awaited. Furthermore, the phase III PIVOTALboost trial will further prospectively evaluate focal dose escalation using IMRT or high-dose-rate (HDR) brachytherapy in patients with intermediate–high risk localized prostate cancer [51]. 

High-risk non-metastatic prostate cancer represents a heterogeneous population with a distinct biology. Accurate risk stratification to guide systemic treatment is essential. Based on the multivariate analysis of clinical outcomes in the current study, a significant association was observed between ISUP grade group 5 disease and inferior RRFS. Moving forward, in the era of precision oncology, apart from the use of clinicopathological factors in risk stratification, the incorporation of molecular biomarkers, such as the 22-gene genomic classifier Decipher score, may potentially improve the prognostication and discrimination of patients with high-risk prostate cancer for individualized systemic treatment strategies [59]. The ongoing NRG-GU009 (PREDICT-RT) study will shed light on the role of genomic risk stratification on the intensification or deintensification of systemic treatment with standard radiation [60].

The advent of modern imaging modalities such as PMSA PET-CT has allowed for the detection of nodal and distant metastases at a superior sensitivity and specificity [39,41]. The United States Food and Drug Administration (FDA) supports the use of PSMA PET-CT in patients exhibiting equivocal metastatic findings or those at a high risk of metastatic disease when conventional imaging yields negative results [61]. With the increase in availability of PSMA PET-CT, it is likely that more patients will present with cN1 disease at initial diagnosis. Nevertheless, it remains unclear whether the alteration in management decisions based on the PSMA PET-CT stage would impact on long-term survival outcomes. In the current study, PSMA PET-CT staging was associated with a trend towards better BRFS, especially in patients with cN0 disease, although this was not statistically significant (*p* = 0.060). This may suggest that undetected occult metastasis using conventional imaging could result in inferior biochemical control when adopting the same definitive treatment strategy. In the real world, the accessibility of PSMA PET-CT is often limited due to cost. Identifying patients who derive the most benefit from sensitive tumor staging may improve the cost effectiveness. In the current study, patients who had ISUP grade group 5 disease and were staged using PSMA PET-CT were associated with significantly better BRFS after definitive RT compared to those staged using conventional imaging. In real-world clinical practice, the adoption of PSMA PET-CT for patients with high-risk features such as ISUP grade group 5 disease or equivocal nodal metastasis may be considered to exclude distant metastasis before standard definitive RT or identify patients for treatment intensification.

The current study was a single-institution retrospective study with limited patient numbers, a relatively short follow-up duration, and lack of patient-reported outcomes. The retrospective nature of this study may have resulted in the under-reporting of low-grade toxicity. A lack of standard local salvage treatment options after biochemical relapse may have led to inconsistent referral patterns for local restaging imaging. Despite these limitations, our study represents one of the largest reports on definitive RT for high-risk and very high-risk locoregional prostate cancer in Chinese patients in the context of contemporary staging and advanced RT techniques, and clinical outcomes from this treatment approach are highly encouraging. Extended follow-ups to verify long-term treatment outcomes and monitor for late toxicities as well as validation from large, prospective multicenter series are highly anticipated. 

## 5. Conclusions

Five-year institutional experience of VMAT for the definitive treatment of high-risk and very high-risk locoregional prostate cancer demonstrates encouraging treatment outcomes and a low incidence of severe treatment-related toxicity. Notably, ISUP grade group 5 and cN1 stage were associated with worse RRFS. Initial staging with PSMA PET-CT compared to conventional imaging was associated with better BRFS in patients with ISUP grade group 5 disease. In clinical practice, initial PSMA PET-CT staging for patients with high-grade disease or suspected nodal involvement and tailored pelvic irradiation based on nodal risk should be considered to maximize the clinical benefits. 

## Figures and Tables

**Table 1:** Clinicopathological and treatment characteristics.

	**Overall (N = 209)**		Roach estimated nodal risk, %; median (IQR)	31.0 (22.3, 43.9)
**Patient characteristics**			NCCN risk group; N (%)	
Age, years; median (IQR)	72 (67, 77)		High-risk cN0	97 (46.4)
ECOG performance status; N (%)			Very high-risk cN0	96 (45.9)
0–1	184 (88.0)		cN1	16 (7.7)
2	25 (12.0)		**Investigations**	
Charlson comorbidity index; N (%)			Prostate biopsy method; N (%)	
≤2	36 (17.2)		Systematic biopsy	163 (78.0)
>2	173 (82.8)		Systematic + targeted biopsy	28 (13.4)
**Clinicopathological characteristics**			Not available	18 (8.6)
Positive core/total core ratio; median (IQR)	0.50 (0.25, 0.80)		Prostate biopsy approach; N (%)	
ISUP grade group 4–5 core/total core ratio; median (IQR)	0.00 (0.00, 0.17)		Transrectal	166 (79.4)
PNI; N (%)			Transperineal	27 (12.9)
Negative	131 (62.7)		Others ^#^	16 (7.7)
Positive	36 (17.2)		Staging imaging; N (%)	
Not available	42 (20.1)		Conventional imaging	120 (57.4)
ISUP grade group; N (%)			PSMA PET-CT	89 (42.6)
1–3	118 (56.5)		**Treatment characteristics**	
4	58 (27.8)		ADT; N (%)	
5	33 (15.8)		2 years	10 (4.8)
T stage; N (%)			3 years	187 (89.5)
1–2	87 (41.6)		>3 years ^^^	10 (4.8)
3a	71 (34.0)		Others	2 (1.0)
3b-4	51 (24.4)		RT coverage; N (%)	
N stage; N (%)			Prostate/SV	48 (23.0)
0	193 (92.3)		Prostate/SV + elective pelvic LN	149 (71.3)
1	16 (7.7)		Prostate/SV + elective pelvic LN + involved LN boost *	12 (5.7)
Baseline PSA, ng/mL; median (IQR)	22.6 (11.4, 42.7)		RT OTT, days; median (IQR)	55 (52, 56)

^#^ Other means of pathological diagnosis include FC and TURP. ^^^ ADT > 3 years includes LHRH analogue (or antagonist) and orchidectomy. * One patient received SIB boost to the dubious LNs. Abbreviations: IQR = interquartile range; ECOG = Eastern Cooperative Oncology Group; ISUP = international Society of Urological Pathology; PNI = perineural invasion; PSA = prostate-specific antigen; LN = lymph node; NCCN = National Comprehensive Cancer Network; PSMA PET-CT = prostate-specific membrane antigen positron emission tomography–computed tomography; ADT = androgen deprivation therapy; RT = radiotherapy; SV = seminal vesicle; OTT = overall treatment time; FC = flexible cystoscopy; TURP = transurethral resection of the prostate; LHRHa = luteinizing hormone-releasing hormone analogue; SIB = simultaneous integrated boost.

**Table 2:** Actuarial estimates for BRFS, RRFS, MFS and PCSS.

	BRFS	RRFS	MFS	PCSS
24 months	93.8%	99.5%	99.5%	100%
36 months	86.9%	99.5%	99.5%	100%
48 months	85.2%	96.8%	96.8%	100%
60 months	85.2%	95.4%	96.8%	98.8%

Abbreviations: BRFS = biochemical relapse-free survival; RRFS = radiographic relapse-free survival; MFS = metastasis-free survival; PCSS = prostate cancer-specific survival.

**Table 3:** Patterns of failure at the time of first radiographic relapse.

	Initial Staging Imaging	Clinical Stage	Baseline PSA, ng/mL	Gleason Score	Positive/ Total Core Ratio	ISUP Group 4–5/Total Core Ratio	ADT Duration, Years	RT Coverage	Nadir PSA, ng/mL	Relapse Pattern	Time to Failure, Months
1	Conventional	T3aN0M0	27.2	4 + 3	0.67	0	3	Prostate/SV + elective pelvic LN	0.06	Prostate	55.6
2	Conventional	T3aN0M0	4.5	4 + 5	0.5	0.42	3	Prostate/SV + elective pelvic LN	<0.02	Pelvic LN, distant LN, bone metastasis	38.8
3	PSMA PET-CT	T3bN1M0	40.9	4 + 3	1	0	3	Prostate/SV + elective pelvic LN	0.08	Bone metastasis	47.6
4	Conventional	T3bN1M0	83.9	4 + 5	0.62	0.15	3	Prostate/SV + elective pelvic LN + involved LN boost	0.23	Bone metastasis	36.3
5	PSMA PET-CT	T2cN1M0	21.7	5 + 4	0.85	0.85	>3	Prostate/SV + elective pelvic LN + involved LN boost	0.29	Bone metastasis	19.5

Abbreviations: PSA = prostate-specific antigen; ISUP = international Society of Urological Pathology; ADT = androgen deprivation therapy; RT = radiotherapy; SV = seminal vesicle; LN = lymph node; PSMA PET-CT = prostate-specific membrane antigen positron emission tomography–computed tomography.

**Table 4:** Comparison of radiotherapy related toxicities across different RT coverage.

			Overall (N = 209)	RT Coverage		
				Prostate/SV (N = 48)	Prostate/SV + Pelvis (N = 149)	Prostate/SV + Pelvis + Boost (N = 12)
Acute toxicity; N (%)	GU	Grade 1	29 (13.9)	10 (20.8)	17 (11.4)	2 (16.7)
		Grade 2	49 (23.4)	12 (25.0)	37 (24.8)	0 (0.0)
		Grade 3	15 (7.2)	4 (8.3)	9 (6.0)	2 (16.7)
	GI	Grade 1	75 (35.9)	13 (27.1)	57 (38.3)	5 (41.7)
		Grade 2	18 (8.6)	1 (2.1)	17 (11.4) *	0 (0.0)
		Grade 3	0 (0.0)	0 (0.0)	0 (0.0)	0 (0.0)
Late toxicity; N (%)	GU	Grade 1	11 (5.3)	4 (8.3)	5 (3.4)	2 (16.7)
		Grade 2	29 (13.9)	6 (12.5)	22 (14.8)	1 (8.3)
		Grade 3	4 (1.9)	2 (4.2)	1 (0.7)	1 (8.3)
	GI	Grade 1	42 (20.1)	9 (18.8)	32 (21.5)	1 (8.3)
		Grade 2	19 (9.1)	3 (6.3)	15 (10.1)	1 (8.3)
		Grade 3	4 (1.9)	2 (4.2)	2 (1.3)	0 (0.0)
	Sacral insufficiency fracture		1 (0.5)	0 (0.0)	0 (0.0)	1 (8.3)

* *p* = 0.079, prostate/SV vs. prostate/SV + pelvis groups. Abbreviations: RT = radiotherapy; SV = seminal vesicle; GU = genitourinary; GI = gastrointestinal.

**Table 5:** Clinicopathological characteristics by RT coverage for cN0 patients.

	RT Coverage		
	Prostate/SV (N = 48)	Prostate/SV + Pelvis (N = 145)	*p*
Positive core/total core ratio; median (IQR)	0.42 (0.17, 0.58)	0.50 (0.25, 0.83)	0.015
ISUP grade group 4–5 core/total core ratio; median (IQR)	0.00 (0.00, 0.00)	0.00 (0.00, 0.20)	0.019
PNI; N (%)			0.457
Negative	12 (25.0)	28 (19.3)	
Positive	31 (64.6)	92 (63.4)	
Not available	5 (10.4)	25 (17.2)	
ISUP grade group; N (%)			0.063
1–3	35 (72.9)	78 (53.8)	
4	8 (16.7)	46 (31.7)	
5	5 (10.4)	21 (14.5)	
T stage; N (%)			0.089
1–2	16 (33.3)	67 (46.2)	
3a	23 (47.9)	44 (30.3)	
3b-4	9 (18.8)	34 (23.4)	
Baseline PSA, ng/mL; median (IQR)	13.5 (8.2, 22.0)	25.2 (15.4, 43.1)	0.051
Roach estimated nodal risk, %; median (IQR)	15.7 (13.8, 33.3)	33.4 (24.5, 43.9)	0.004
NCCN risk group; N (%)			0.134
High-risk cN0	29 (60.4)	68 (46.9)	
Very high-risk cN0	19 (39.6)	77 (53.1)	
ADT; N (%)			0.865
2 years	3 (6.3)	7 (4.8)	
3 years	44 (91.7)	134 (92.4)	
>3 years ^^^	1 (2.1)	2 (1.4)	
Others	0 (0.0)	2 (1.4)	

^^^ ADT > 3 years includes LHRH analogue (or antagonist) and orchidectomy. Abbreviations: RT = radiotherapy; LN = lymph node; SV = seminal vesicle; IQR = interquartile range; ISUP = International Society of Urological Pathology; PNI = perineural invasion; PSA = prostate-specific antigen; NCCN = National Comprehensive Cancer Network; ADT = androgen deprivation therapy.

**Table 6:** Clinical outcomes stratified by clinicopathological and investigation variables.

		BRFS		RRFS	
		*p* *	HR (95% CI) *	*p* *	HR (95% CI) *
*Clinicopathological characteristics*					
Positive core/total core ratio	Continuous variable	0.465		0.080	18.335 (0.705–476.549)
ISUP grade group 4–5 core/total core ratio	Continuous variable	0.603		0.277	
PNI	Negative	0.672		0.209	
	Positive				
ISUP grade group	1–3	0.386		0.078	Reference
	4			0.975	
	5			**0.024**	**7.939 (1.313–48.001)**
T stage	1–2	0.903		0.616	
	3a				
	3b-4				
N stage	0	0.186		**0.000**	**Reference**
	1				**42.508 (6.495–278.201)**
Baseline PSA, ng/mL	Continuous variable	0.837		0.585	
Roach estimated nodal risk, %	Continuous variable	0.424		0.157	
*Investigations*					
Prostate biopsy method	Systematic biopsy	0.951		0.087	Reference
	Systematic + targeted biopsy				4.815 (0.795–29.173)
Staging imaging	Conventional imaging	0.083	Reference	0.632	
	PSMA PET-CT		0.428 (0.159–1.150)		

* denote univariate analysis. Bold values denote statistical significance with *p* < 0.05 level in the multivariate analysis. Abbreviations: BRFS = relapse-free survival; RRFS = radiographic relapse-free survival; HR = hazard ratio; CI = confidence interval; ISUP = international Society of Urological Pathology; PSA = prostate-specific antigen; LN = lymph node; PSMA PET-CT = prostate-specific membrane antigen positron emission tomography–computed tomography.

**Table 7:** Impact of PET-CT on BRFS, accounting for interaction ^^^ with other clinicopathological variables.

		BRFS
		*p* *
Positive core/total core ratio	Continuous variable	0.325
ISUP grade group 4–5 core/total core ratio	Continuous variable	0.096
PNI	Categorial variable	0.908
	Negative	
	Positive	
	Not available	
ISUP grade group	Categorial variable	0.039
	1–3	Reference
	4	HR * 0.130 95% CI * 0.006–3.001
	5	HR * 0.010 95% CI * 0.000–0.367
T stage	Categorial variable	0.630
	1–2	
	3a	
	3b-4	
Baseline PSA, ng/mL	Continuous variable	0.098

^^^ Including an interaction term for the staging modality (conventional imaging vs. PSMA PET-CT) and each clinicopathological variable, and the main terms of the staging modality and clinicopathological variables of positive core/total core ratio, ISUP grade group 4–5 core/total core ratio, PNI, ISUP grade group, T stage, baseline PSA, Roach estimated nodal risk and NCCN risk group to the Cox proportional hazards model. * *p* value, HR and 95% CI for the interaction term. Abbreviations: BRFS = biochemical relapse-free survival; ISUP = International Society of Urological Pathology; PNI = perineural invasion; PSA = prostate-specific antigen; HR = hazard ratio; CI = confidence interval; PSMA PET-CT = prostate-specific membrane antigen positron emission tomography–computed tomography; LN = lymph node; NCCN = National Comprehensive Cancer Network.

**Table 8:** Comparison of patient characteristics and outcomes of randomized trials and the current study.

	NHT + WPRT Arm in RTOG 9413 [16,17,18]	WPRT Arm in GETUG-01 [27,48]	WPRT Arm in POP-RT [19]	Elective Pelvic RT in the Current Study
Median age	Not specified	69.8 years	66 years	71 years
Roach estimated nodal risk	≥15%	48.7% patients ≥ 15%	≥20%	≥15%
Baseline PSA, ng/mL	33% patients with PSA > 30	Median 12	Median 29.9	Median 25.5
GS	74% patients with GS 7–10	12.5% patients with GS 8–10	48.1% patients with GS 8–10	45.8% patients with GS 8–10
Clinical T stage	67% patients with T2c-T4	24.2% patients with cT3	74.6% patients with cT3–4	53.5% patients with cT3–4
Staging modality	Conventional	Conventional	Conventional and/or PSMA PET-CT (80%)	Conventional and/or PSMA PET-CT (42%)
ADT duration	4 months	6 months	≥2 years (14.5% orchidectomy)	3 years
RT technique	Conventional 2-D box	Conventional 2-D box and 3-D conformal	IMRT or tomotherapy	VMAT
Dose and fractionation to prostate (BED)	70.2 Gy in 1.8-Gy fractions (112.32 Gy)	66–70 Gy in 1.8 to 2-Gy fractions (110–122 Gy)	68 Gy in 2.72-Gy fractions (129.6 Gy)	76 Gy in 2-Gy fractions (126.7 Gy)
Dose and fractionation to whole pelvis	50.4 Gy in 1.8-Gy fractions	46 Gy in 2-Gy fractions	50 Gy in 2-Gy fractions	46 Gy in 2-Gy fractions
Definition of biochemical failure	Phoenix criteria	ASTRO definition	Phoenix criteria	ASTRO definition
Median follow-up	8.8 years	11.4 years	5.7 years	4 years
Primary outcome	10-year PFS 28.4%	5-year EFS 69.2%	5-year BFFS 95%	4-year BRFS 87%
Other clinical outcome estimates	Not specified	Not specified	5-year	4-year
			DFS 89.5%	BRFS 87%
			MFS 95.9%	RRFS 99%
				MFS 99%
				PCSS 100%
Grade ≥ 3 late toxicities				
GU toxicity	6%	15.3%	1.8%	0.7%
GI toxicity	7%	10.7%	1.8%	1.4%

Abbreviations: NHT = neoadjuvant hormonal therapy; WPRT = whole pelvic radiotherapy; RTOG = Radiation Therapy Oncology Group; GETUG = Urogenital Tumor Study Group; RT = radiotherapy; PSA = prostate-specific antigen; GS = Gleason score; PSMA PET-CT = prostate-specific membrane antigen positron emission tomography–computed tomography; ADT = androgen deprivation therapy; IMRT = intensity-modulated radiation therapy; VMAT = volumetric modulated arc therapy; BED = biologically effective dose; ASTRO = American Society for Radiation Oncology; PFS = progression-free survival; EFS = event-free survival; BFFS = biochemical failure-free survival; BRFS = biochemical relapse-free survival; DFS = disease-free survival; MFS = metastasis-free survival; RRFS = radiographic relapse-free survival; PCSS = prostate cancer-specific survival; GU = genitourinary; GI = gastrointestinal.

## Data Availability

The data presented in this study are available in an anonymized form upon request from the corresponding author.

## Appendix A. Planning Objectives and Dose Specifications for Tumor Targets and Organs at Risk

**Table:**

PTV	D2% ≤ 105% (variation acceptable: ≤ 107%)
	D99% ≥ 95% (variation acceptable: ≥ 93%)
	D98% ≥ 100% (variation acceptable: ≥ 95%)
CTV	D99% ≥ 100% (variation acceptable: ≥ 95%)
PTVnx	D98% ≥ 95%
CTVnx	D98% ≥ 100%
Rectum	V70 ≤ 20%
	V50 ≤ 50%
Bladder	V70 ≤ 30%
	V55 ≤ 50%
Bowel	D0.03cc ≤ 52.5 Gy (variation acceptable: ≤ 54 Gy)
Femoral heads	V50 ≤ 5%

Abbreviations: PTV = planning target volume; CTV = clinical target volume.

## List of Abbreviations:

Androgen deprivation therapy	ADT
American Joint Committee on Cancer	AJCC
American Society for Radiation Oncology	ASTRO
Biochemical failure-free survival	BFFS
Biochemical relapse-free survival	BRFS
Confidence interval	CI
Disease-free survival	DFS
Equivalent dose at 2 Gy per fraction	EQD2
Eastern Cooperative Oncology Group	ECOG
Event-free survival	EFS
Food and Drug Administration	FDA
Genitourinary	GU
Gastrointestinal	GI
Hazard ratio	HR
High dose rate	HDR
Intensity-modulated radiation therapy	IMRT
Image-guided radiotherapy	IGRT
Interquartile range	IQR
International Society of Urological Pathology	ISUP
Institutional Review Board	IRB
Luteinizing hormone-releasing hormone analogue	LHRHa
Metastasis-free survival	MFS
Magnetic resonance imaging	MRI
National Comprehensive Cancer Network	NCCN
Overall survival	OS
Organs at risk	OARs
Overall treatment time	OTT
Prostate cancer-specific survival	PCSS
Prostate-only radiotherapy	PORT
Prostate-specific membrane antigen positron emission tomography–computed tomography	PSMA PET-CT
Primary Tumor Clinical Target Volume	CTVp
Prostate-specific antigen	PSA
Progression-free survival	PFS
Radiotherapy	RT
Radiation Therapy Oncology Group	RTOG
Radiographic relapse-free survival	RRFS
Simultaneous integrated boost	SIB
Stereotactic body radiation therapy	SBRT
Transperineal	TP
Volumetric modulated arc therapy	VMAT
Whole pelvic radiotherapy	WPRT
